# Comparative efficacy of different single drugs to prevent necrotizing enterocolitis in preterm infants: an update systematic review and network meta-analysis

**DOI:** 10.3389/fnut.2024.1452338

**Published:** 2024-09-09

**Authors:** Jing Chen, Xiao Chen, Xiaoling Huang, Jia Liu, Qingfeng Yu

**Affiliations:** ^1^Department of Neonatology, The First People’s Hospital of Neijiang, Neijiang, China; ^2^Department of Orthopedics, The First People’s Hospital of Neijiang, Neijiang, China

**Keywords:** preterm infants, necrotizing enterocolitis, drugs, network meta-analysis, randomized controlled trials

## Abstract

**Objective:**

To investigate an optimal regimen of six drugs, including lactoferrin, probiotics, prebiotics, glutamine, arginine and erythropoietin (EPO), for the prevention of necrotizing enterocolitis (NEC) in preterm infants.

**Methods:**

PubMed, Embase, Ovid, The Cochrane Library, and Web of Science databases were searched for randomized controlled trials (RCTs) investigating the efficacy of lactoferrin, probiotics, prebiotics, glutamine, arginine, and EPO in preventing NEC in preterm infants, with a cutoff date of June 20, 2024. Two authors independently screened studies and extracted all the data. Network meta-analysis (NMA) was conducted to compare the outcomes of different interventions, and group rankings were determined using the surface under the cumulative ranking curve (SUCRA).

**Results:**

A total of 89 RCTs with 26,861 preterm infants were included. Arginine demonstrated the highest clinical efficacy in reducing the incidence of NEC, with probiotics being the next most effective and the placebo being the least effective. Lactoferrin was identified as the most effective intervention for reducing the incidence of NEC-associated sepsis. Prebiotics showed the highest effect on overall mortality, reducing the beginning of enteral feeding, and were associated with the shortest hospital stay. Glutamine significantly decreased the time to full enteral feeding.

**Conclusion:**

Existing literature highlights arginine as the most efficacious pharmacological agent in preventing NEC in preterm infants. It has been shown to effectively lower the rates of NEC, septicemia, and mortality, warranting its recommendation as the first-line clinical intervention. Following this, probiotics are recommended as a second option.

## 1 Introduction

Necrotizing enterocolitis (NEC) is among the most prevalent critical conditions affecting premature infants (1–3), found in 5–12% of very low birth weight (VLBW) infants (4–6). It presents with necrosis of the intestinal tissues in small and large bowels, which leads to a translocation of gut microbiota into the bloodstream and can also lead to sepsis (7–10). In general, in stage II, or definitive disease, there is nearly always evidence for pneumatosis intestinalis and/or portal venous gas (3, 11). Mortality rates among neonates requiring surgery are estimated to be 20–30% (3). Beyond the high mortality, NEC also carries a significant risk of morbidity in survivors, manifesting as short bowel syndrome and developmental stagnation (12). The complexity of NEC lies in its resistance to intervention once fully established, compounded by the scarcity and expense of treatment options. Use of antibiotics, gastric decompression, and parenteral nutrition are the most common (9). The etiology of NEC remains elusive, with the debate ongoing on whether it constitutes a single pathological entity or a spectrum of related disorders. Despite advancements in deciphering its pathophysiological mechanisms, substantial gaps in knowledge persist, potentially accounting for the stagnant progress in NEC therapeutics over recent decades (13). Consequently, NEC prevention is underscored as a vital strategy to mitigate premature infant mortality and morbidity rates.

Breastfeeding is recognized as a safe and effective preventive approach for NEC in preterm infants (14, 15); yet, the role of other adjunctive medications or additives is also significant. For example, probiotics, prebiotics, glutamine, arginine, lactoferrin, and EPO have been studied as a therapy to decrease the risk of NEC among preterm infants (16–22). While initial data have suggested that probiotics can reduce the incidence and mortality of NEC (23–25), efficacy and potential short-term or long-term side effects of the other therapies remain unclear. Given the unique characteristics of the gastrointestinal (GI) tract in preterm infants, the concurrent use of multiple additives is generally discouraged.

Network meta-analysis (NMA) compares three or more interventions simultaneously in a single analysis by combining direct and indirect evidence across a network of studies (26). The major advantage over traditional meta-analysis is that this approach integrates direct and indirect data, enabling a comprehensive comparison and efficacy ranking of multiple interventions to identify the optimal strategy (27).

This study employed NMA to assess and rank the preventive and therapeutic effects of probiotics, prebiotics, glutamine, arginine, lactoferrin, and EPO on NEC in preterm infants, intending to provide valuable evidence-based medical evidence for drug selection in future clinical practice.

## 2 Methods

### 2.1 Protocol and registration

This study adhered to the Preferred Reporting Items for Systematic Reviews and Meta-Analyses (PRISMA) 2020 statement (28), ensuring a structured methodology and reporting format, and A Measurement Tool to Assess systematic Reviews (AMSTAR) 2 guidelines (29). The NMA protocol has been duly registered in the PROSPERO database (the registration number: CRD42024496947).

### 2.2 Data sources

A comprehensive literature search was conducted independently by two researchers (the first and second authors); disparities were resolved by discussion. The search encompassed titles and abstracts, and full-text assessments were carried out as needed to determine study eligibility.

The following databases were systematically searched from their inception until June 20, 2024: PubMed, Embase, Ovid, The Cochrane Library, and Web of Science. Placebo-controlled and head-to-head RCTs examining probiotics, prebiotics, glutamine, arginine, lactoferrin, and EPO as therapy against NEC in preterm infants were included. The following relevant terms were searched: (“enterocolitis necrotizing [MeSH Terms]” OR “necrotizing enterocolitis”) AND (“lactoferrin” OR “probiotics” OR “prebiotics” OR “glutamine” OR “arginine” OR “erythropoietin”). Additionally, Google Scholar was consulted to identify potentially relevant literature. Furthermore, the reference lists of identified reports were meticulously reviewed to identify any additional pertinent studies. Only articles published in the English language were considered for inclusion. The detailed search strategy is shown in Table 1 (PubMed is used as an example).

**Table 1:** Search strategy on PubMed.

#1	Enterocolitis necrotizing [MeSH Terms]
#2	Enterocolitis necrotizing [Title/Abstract]
#3	#1 OR #2
#4	Lactoferrin [MeSH Terms]
#5	Lactoferrin [Title/Abstract]
#6	Probiotics [MeSH Terms]
#7	Probiotics [Title/Abstract]
#8	Prebiotics [MeSH Terms]
#9	Prebiotics [Title/Abstract]
#10	Glutamine [MeSH Terms]
#11	Glutamine [Title/Abstract]
#12	Arginine [MeSH Terms]
#13	Arginine [Title/Abstract]
#14	Erythropoietin [MeSH Terms]
#15	Erythropoietin [Title/Abstract]
#16	#4 OR #5 OR #6 OR #7 OR #8 OR #9 OR #10 OR #11 OR #12 OR #13 OR #14 OR #15
#17	# 3 AND #16

### 2.3 Eligibility criteria

The inclusion criteria were as follows: (1) participants: Preterm infants born <34 weeks of gestation and/or infants with birth weight < 1,500 g; (2) types of studies: RCTs; (3) interventions: administration of early lactoferrin, probiotics, prebiotics, glutamine, arginine, erythropoietin and placebo (< 8 days of postnatal age) by any route and dose continued for any duration; each study involved at least two interventions; (4) Outcomes: primary outcomes: the incidence of NEC, NEC-associated sepsis and overall mortality; secondary outcomes: time to beginning enteral feeds, time to full enteral feeds and duration of hospitalization.

The exclusion criteria were: (1) non-RCTs, including quasi-RCTs, case–control studies, cohort studies, case reports, protocols, review articles, meta-analyses, editorials, letters, animal studies, cadaveric trials, or conference abstracts; (2) studies with <20 cases; (3) studies combining drugs (e.g., a combination of lactoferrin and probiotics); (4) poor-quality research literature or studies lacking rigor in their design; (5) duplicate or similar documents published by the same author in different journals; (6) incomplete data or important research data could not be obtained through email and other contacts; (7) non-English articles.

### 2.4 Data extraction

A specifically designed form was employed to extract essential information from each study. The following data were extracted: (1) general information such as the lead author, year of publication, study design, and country in which the study was performed; (2) demographic information, including the number and proportion of male or female infants, gestational age, birth weight, and the number of infants involved; (3) details regarding the drugs (intervention and comparison); (4) information on clinical outcomes, including the incidence of NEC, NEC-associated sepsis, overall mortality, beginning enteral feeding (time), full enteral feeding (time), and duration of hospitalization. In instances where SD was not available from the publication, SD was imputed using the method prescribed in the Cochrane Handbook, as follows:

1. Obtaining SDs for a group of means were calculated from standard error of the mean (SEM) or 95% confidence intervals (CIs) by using equations from the Cochrane Handbook chapter 6.5.2.2 when the group SDs were not provided directly;

$$\left[\text{SD}=\text{SEM}\times \sqrt{n}\;\text{or}\;\text{SD}=\sqrt{n}\times \left(\text{upperlimit}-\text{lowerlimit}\right)/3.92\right]$$

2. When concentrations were provided in medians and 25th – 75th percentile, we converted these into means ± SD by using the equation developed by Wan et al. (Cochrane Handbook chapter 6.5.2.5);
3. when not reported, change-from-baseline SDs were estimated using the equation developed by Follmann et al., assuming a correlation coefficient of 0.50 between baseline and post-intervention lipid and lipoprotein values [Cochrane Handbook chapter 6.5.2.8, 2].

$$\left[\begin{array}{l} SD_{E,\text{change}}=\sqrt{SD_{E,\text{change}}^{2}}+SD_{E,\text{change}}^{2}-\\ \left(2\times 0.50\times SD_{E,\text{baseline}}\times SD_{E,\text{final}}\right) \end{array}\right]$$

### 2.5 Quality assessment

The Cochrane Risk of Bias Tool was employed to assess the quality. The risk of bias for the included trials was evaluated by two researchers based on the Cochrane Handbook criteria. The criteria covered randomization, allocation concealment, blinding of participants and personnel, blinding of outcome assessors, completeness of outcome data, selective reporting, and other biases. Each domain was classified as having an unclear risk, low risk, or high risk of bias. The assessment was deemed to be of high quality if most of the domains were well-described and exhibited a low risk of bias. In cases of discrepancies in the ratings, researchers reached a consensus through discussion.

### 2.6 Statistical analysis

To conduct a comprehensive NMA, we utilized the statistical software packages “Network” and “mvmeta” within STATA 17.0 software. Dichotomous variables, specifically the incidence of NEC, NEC-associated sepsis and overall mortality, were analyzed using relative risk (RR) with corresponding 95% confidence intervals (CI). Meanwhile, continuous variables, including time to beginning enteral feeds, time to full enteral feeds and duration of hospitalization, were analyzed using weighted mean differences (WMD) with corresponding 95% CI. The comparison was considered statistically non-significant when the 95% CI of the RR or WMD contained the value 1.

For direct comparisons, a conventional meta-analysis was conducted to aggregate the results using random-effects models, serving as sensitivity analyses. NMA employed a frequentist approach with a random-effects model to estimate direct and indirect comparisons. The primary objective of the NMA was to assess whether comparator interventions demonstrated superiority. Global inconsistency, local inconsistency (using a node-splitting approach), and loop inconsistency were used to evaluate potential inconsistencies between indirect and direct comparisons. Statistical significance for global inconsistency was determined using *p*-values, with *p* > 0.05 indicating no significant global inconsistency. Local inconsistency was assessed through node-splitting analysis, and p > 0.05 indicated no significant local inconsistency. Heterogeneity within each closed loop was estimated using the inconsistency factor (IF), with a 95% CI (IF) value of zero signifying no statistical significance. A global network diagram was employed in each pre-specified outcome to illustrate direct comparisons between interventions. The size of the nodes in the diagram corresponded to the number of participants receiving each treatment. Lines linked treatments subject to direct comparisons, and the thickness of these lines was proportional to the number of trials evaluating the specific comparison.

Within the “Results” section, the ranking probability of each intervention was presented using a cumulative probability ranking graph. The graph incorporated the Surface Under the Cumulative Ranking Curve (SUCRA) value, serving as an index summarizing the cumulative ranking probability. The SUCRA value ranged between 0 and 100%, where a larger SUCRA value indicated a higher ranking for the intervention, typically reflecting a more favorable or less favorable effect. All intervention measures were ranked based on their respective SUCRA values or the area under the curve, resulting in a comprehensive ranking of the interventions.

A comparison-adjusted funnel plot was used to assess the potential for publication bias. This analysis aimed to determine whether there was evidence of a small sample effect or publication bias within the intervention network.

## 3 Results

### 3.1 Search results

**Figure 1:**
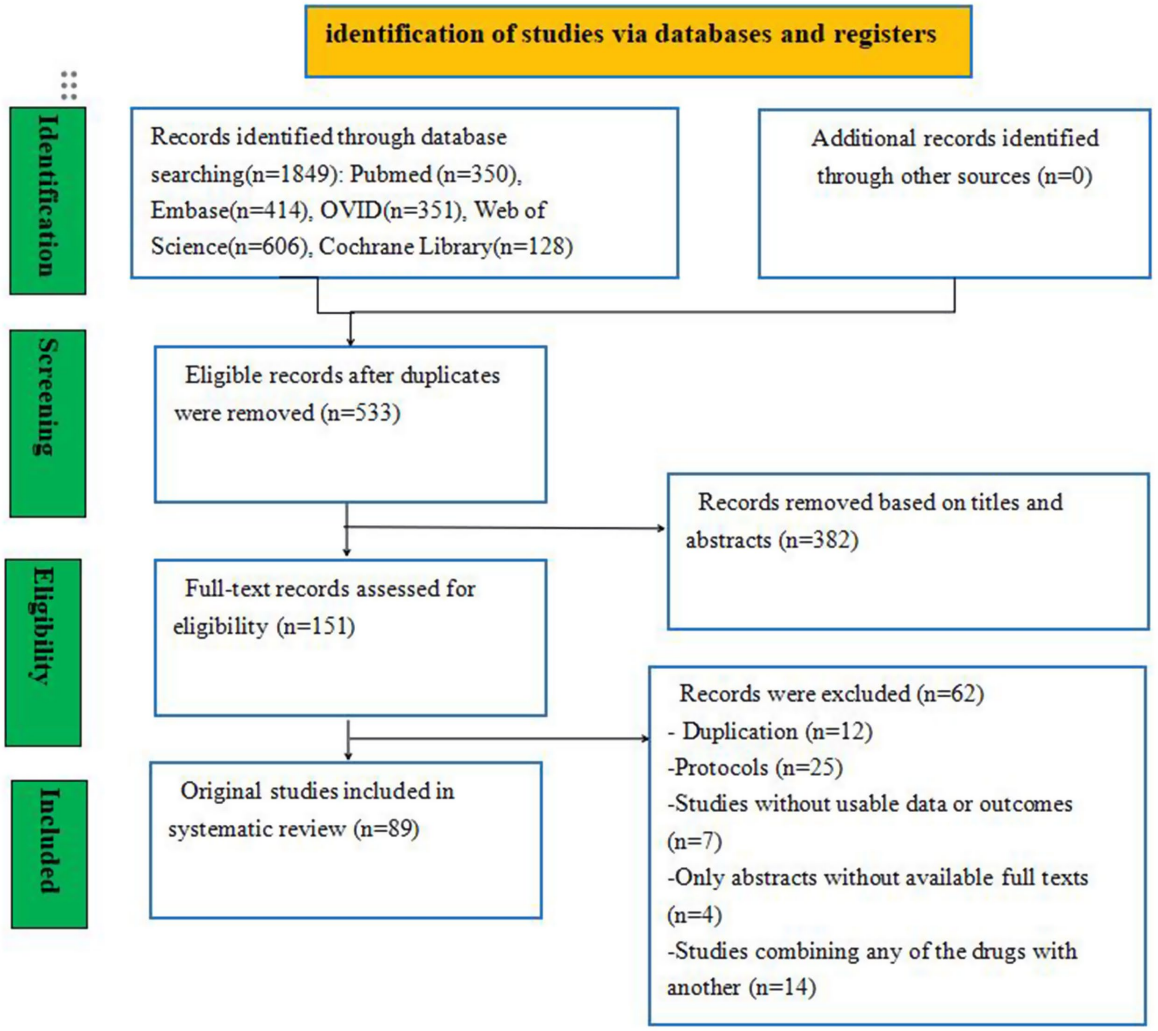
Flow diagram of the study selection process.

A total of 23,357 studies were initially identified, including PubMed (*n* = 350), Embase (*n* = 414), Ovid (*n* = 351), Web of Science (*n* = 606), and the Cochrane Library (*n* = 128) studies. To eliminate duplicate entries, the “Find duplicates” function in EndNote software was employed, removing 1,316 studies. After thoroughly screening titles and abstracts, 382 irrelevant references were excluded. Subsequently, a full text was retrieved for the remaining 151 references. Ultimately, 89 studies involving 26,861 neonates met the eligibility criteria for inclusion in this NMA. The study selection process is illustrated in Figure 1, and the baseline characteristics of the included studies are summarized in Table 2.

**Table 2:** Baseline characteristics of the included studies.

Author	Country	Study design	Group	NO	Gestational age (week)	Birth weight (g)	Outcome
Akin 2014	Turkey	RCTs	Lactoferrin	22	29.5 ± 1.6	1,290 ± 346.7	(1)(2)(3)
			Placebo	23	30.3 ± 2.5	1,307 ± 262.1	
Al-Hosni 2012	United States	RCTs	Probiotic	50	25.7 ± 1.4	778 ± 138	(1)(2)(3)
			Placebo	51	25.7 ± 1.4	779 ± 126	
Amin 2002	Canada	RCTs	L-arginine	75	27.4 ± 0.3	952 ± 25	(1)(2)
			Placebo	77	27.6 ± 0.2	955 ± 20	
Armanian 2014	Iran	RCTs	Prebiotic	25	30.48 ± 2.31	1262.80 ± 213.35	(1)(2)(3)(4)(5)(6)
			Placebo	50	30.38 ± 2.53	1205.60 ± 177.23	
Barrington 2016	Canada	RCTs	Lactoferrin	40	28.0 ± 1.7	1,087 ± 315	(1)(2)(3)
			Placebo	39	28.4 ± 2.1	1,104 ± 320	
Bierer 2006	United States	RCTs	EPO	7	26.0 ± 1.1	752 ± 150	(1)(3)
			Placebo	9	26.9 ± 2.1	801 ± 103	
Bin nun 2005	Israel	RCTs	Probiotic	72	29.8 ± 2.6	1,152 ± 262	(1)(2)(3)(4)(5)
			Placebo	73	29.3 ± 4.3	1,111 ± 278	
Braga 2012	Brazil	RCTs	Probiotic	119	29.5 ± 2.5	1194.7 ± 206.3	(1)(2)(3)(4)(5)
			Placebo	112	29.2 ± 2.6	1151.4 ± 224.9	
Chang 2022	China	RCTs	Probiotic	70	26.0 (25.0–27.0)	780.0 (689.3–915.0)	(1)(2)(3)(4)(5)(6)
			Placebo	50	26.0 (25.0–27.0)	815.0 (757.5–920.0)	
Chaudhuri 2014	India	RCTs	Probiotic	56	32 ± 2	1,192 ± 341	(1)(2)(3)(5)(6)
			Placebo	56	32 ± 2	1,069 ± 365	
Chou 2010	China	RCTs	Probiotic	153	28.5 ± 2.3	1103.6 ± 232.4	(1)(2)(3)(6)
			Placebo	148	28.5 ± 2.3	1097.2 ± 231.4	
Costalos 2003	Greece	RCTs	Probiotic	51	31.1 (2.5%)	1,651 (470%)	(1)(2)(5)
			Placebo	36	31.8 (2.7%)	1,644 (348.7%)	
Costeloe 2016	United Kingdom	RCTs	Probiotic	650	28.0 (26.1–29.4)	1,039 ± 312	(1)(2)(3)
			Placebo	660	28.0 (26.1–29.6)	1,043 ± 317	
Cui 2019	China	RCTs	Probiotic	45	32.85 ± 1.39	1,682 ± 109.03	(1)(2)(6)
			Placebo	48	32.56 ± 1.41	1714 ± 127.11	
Dallas 1998	United States	RCTs	Glutamine	34	24–32	500–1,250	(6)
			Placebo	33	24–32	500–1,250	
Dani 2002	Italy	RCTs	Probiotic	295	30.8 ± 2.4	1,325 ± 361	(1)(2)(4)
			Placebo	290	30.7 ± 2.3	1,345 ± 384	
Dekieviet 2014	Netherlands	RCTs	Glutamine	30	29.7 ± 1.6	1,270 ± 370	(1)
			Placebo	35	29.0 ± 1.6	1,200 ± 330	
Dilli 2015	Turkey	RCTs	Probiotic	100	28.8 ± 1.9	1,236 ± 212	(1)(2)(3)(6)
			Prebiotic	100	29.0 ± 1.7	1,229 ± 246	
			Placebo	100	28.2 ± 2.2	1,147 ± 271	
El-Ganzoury 2014	Egyp	RCTs	EPO	20	30.2 ± 1.8	1,310 ± 310	(1)(3)(4)(6)
			Placebo	30	30.5 ± 1.5	1,360 ± 290	
El-Shimi 2015	Egypt	RCTs	L-Arginine	25	31.84 ± 2.29	1,450 ± 260	(1)(3)(4)
			Glutamine	25	31.68 ± 1.35	1,450 ± 210	
			Placebo	25	30.64 ± 2.34	1,310 ± 250	
Fauchere 2015	Germany	RCTs	EPO	229	29.0 ± 1.0	1,207 ± 322	(1)(6)
			Placebo	214	29.0 ± 1.0	1,215 ± 365	
Fauchere 2008	Germany	RCTs	EPO	30	28.0 ± 2.0	1,112 ± 347	(1)(2)(3)(6)
			Placebo	15	28.0 ± 2.0	1,081 ± 354	
Fernandez 2012	México	RCTs	Probiotic	75	31.2 (26–35.4)	1,090 (580–1,495)	(1)(3)(6)
			Placebo	75	31 (27–36)	1,170 (540–1,492)	
Fujii 2006	Japan	RCTs	Probiotic	11	31.3 ± 3.16	1,378 ± 365	(1)(6)
			Placebo	8	31.2 ± 1.98	1,496 ± 245	
Griffiths 2018	United Kingdom	RCTs	Lactoferrin	1,098	< 32	1125.9 ± 356.2	(1)(2)(3)(6)
			Placebo	1,101	< 32	1143.3 ± 367.1	
Haiden 2004	Austria	RCTs	EPO	21	25 (23–31)	690 (500–800)	(1)(3)(6)
			Placebo	19	25 (23–28)	690 (467–783)	
Hays 2015	France	RCTs	Probiotic	145	29.0 (28.1–30.1)	1,170 (1000–1,320)	(1)
			Placebo	52	29.4 (27.9–30.6)	1,170 (1055–1,370)	
Hoyos 1999	Colombia	RCTs	Probiotic	918	< 37	Not mentioned	(1)(2)(3)
			Placebo	935	< 37	Not mentioned	
Jacobs 2013	Australia	RCTs	Probiotic	548	27.9 ± 2.0	1,063 ± 259	(1)(2)(3)(5)(6)
			Placebo	551	27.8 ± 2.0	1,048 ± 260	
Juul 2020	United States	RCTs	EPO	476	29.1 ± 6.2	806.4 ± 194.6	(1)(2)(3)
			Placebo	470	28.8 ± 6.2	792.9 ± 182.2	
Kaban 2019	Italy	RCTs	Probiotic	47	33 (28–34)	1,520 (1035–1800)	(1)(2)(3)(6)
			Placebo	47	33 (28–34)	1,605 (1060–1800)	
Kanic 2015	Slovenia	RCTs	Probiotic	40	28.0(27.0–30.0)	1104.1 ± 233.2	(1)(2)(3)(6)
			Placebo	40	29.0 (26.2–30.0)	1024.3 ± 249.9	
Lacey 1996	United States	RCTs	Glutamine	22	26 ± 2	811 ± 175	(5)(6)
			Placebo	22	26 ± 1	800 ± 155	
Lin 2005	China	RCTs	Probiotic	180	28.5 ± 2.5	1,104 ± 242	(1)(2)(3)
			Placebo	187	28.2 ± 2.5	1,071 ± 243	
Lin 2008	China	RCTs	Probiotic	217	<34	1028.9 ± 246.0	(1)(2)(3)(5)
			Placebo	217	<34	1077.3 ± 214.4	
Lowe 2017	United States	RCTs	EPO	35	27.37 ± 1.74	500–1,250	(1)(3)
			Placebo	14	27.64 ± 1.52	500–1,250	
Maier 2002	Germany	RCTs	EPO	68	26 (25–28)	778 (660–880)	(1)
			Placebo	62	27 (26–28)	800 (715–885)	
Manzoni 2006	Italy	RCTs	Probiotic	39	29.6 ± 5	1,212 ± 290	(1)(2)(3)(5)
			Placebo	41	29.3 ± 4	1,174 ± 340	
Manzoni 2009	Italy	RCTs	Lactoferrin	153	29.6 ± 2.5	1,142 ± 244	(1)(2)(3)(5)
			Placebo	168	29.5 ± 3.2	1,109 ± 269	
Manzoni 2014	Italy	RCTs	Lactoferrin	247	29.7 ± 2.5	1,158 ± 251	(1)(3)(5)
			Placebo	258	29.6 ± 2.8	1,118 ± 259	
Mihatsch 2010	Germany	RCTs	Probiotic	91	26.6 ± 1.8	856 ± 251	(1)(3)
			Placebo	89	26.7 ± 1.7	871 ± 287	
Modi 2010	United Kingdom	RCTs	Prebiotic	73	31 (29–32)	1,565 (1350–1880)	(1)(2)
			Placebo	81	30 (28–31)	1,515 (1247–1788)	
Mohamad 2011	Malaysia	RCTs	Glutamine	132	Not mentioned	2,150 ± 910	(1)(2)(3)
			Placebo	138	Not mentioned	2,220 ± 940	
Hosseini 2019	Iran	RCTs	EPO	50	28.7 ± 2.6	1065.1 ± 189.4	(1)(2)(3)
			Placebo	50	27.7 ± 1.5	998.1 ± 172.9	
Nandhini 2015	India	RCTs	Probiotic	108	31.6 ± 1.4	1,430 ± 209	(1)(2)(3)
			Placebo	110	31.4 ± 1.4	1,444 ± 217	
Natalucci 2016	Switzerland	RCTs	EPO	191	29.2 ± 1.6	1,220 ± 327	(1)(2)(6)
			Placebo	174	29.3 ± 1.6	1,213 ± 357	
Obladen 1991	United Kingdom	RCTs	EPO	43	30 ± 1	1,380 ± 324	(1)(3)
			Placebo	50	30 ± 1	1,295 ± 323	
Ochoa 2020	United States	RCTs	Lactoferrin	209	30.8 ± 2.8	1,382 ± 371	(1)(2)(3)(4)(5)
			Placebo	205	30.8 ± 3.2	1,378 ± 353	
O’Gorman 2015	Switzerland	RCTs	EPO	24	30.17 ± 1.44	1,337 ± 332	(1)(2)
			Placebo	34	29.5 ± 1.44	1,192 ± 10	
Ohls 2013	United States	RCTs	EPO	32	27.8 ± 1.9	957 ± 212	(1)(3)(6)
			Placebo	30	27.3 ± 1.8	933 ± 221	
Ohls 2001	United States	RCTs	EPO	59	29 ± 2	1,130 ± 70	(1)(2)(3)(6)
			Placebo	59	28 ± 2	1,118 ± 72	
Ohls 2004	United States	RCTs	EPO	51	26.3 ± 2.0	801 ± 139	(1)(2)
			Placebo	51	25.8 ± 1.7	783 ± 112	
Omar 2020	Egypt	RCTs	EPO	36	32 (31.00–32.00)	Not mentioned	(1)(3)
			Placebo	36	32 (30.50–32.00)	Not mentioned	
Oncel 2013	Turkey	RCTs	Probiotic	200	28.2 ± 2.4	1,071 ± 274	(1)(2)(3)(5)(6)
			Placebo	200	27.9 ± 2.5	1,048 ± 298	
Shannon 1995	United States	RCTs	EPO	77	26.8 ± 1.6	923 ± 184	(1)(2)(3)
			Placebo	80	27.1 ± 1.7	925 ± 183	
Demirel 2013	Turkey	RCTs	Probiotic	135	29.4 ± 2.3	1,164 ± 261	(1)(2)(3)(5)
			Placebo	136	29.2 ± 2.5	1,131 ± 284	
Dutta 2015	India	RCTs	Probiotic	114	30.64 ± 1.64	1286.08 ± 264.76	(1)(2)(3)
			Placebo	35	30.82 ± 1.72	1252.27 ± 309.31	
Güney-Varal 2017	Turkey	RCTs	Probiotic	70	29.7 ± 1.9	1728.5 ± 257	(1)(2)(3)(6)
			Placebo	40	29.3 ± 1.7	1,228 ± 249	
Singh S 2017	Austria	RCTs	Probiotic	37	32.6 ± 2.2	<2000	(1)
			Placebo	35	32.6 ± 2.2	<2000	
Patole 2014	Australia	RCTs	Probiotic	77	29 (26–30)	1,090 (755–1,280)	(1)(2)(5)(6)
			Placebo	76	28 (26–29)	1,025 (810–1,260)	
Peltoniemi 2017	India	RCTs	EPO	21	28.3 ± 1.6	1,141 ± 230	(1)(3)
			Placebo	18	28.2 ± 1.8	1,169 ± 220	
Poindexter 2004	United States	RCTs	Glutamine	721	26.0 ± 2.1	770 ± 141	(1)(2)(3)(6)
			Placebo	712	25.9 ± 1.9	768 ± 138	
Polycarpou 2013	United States	RCTs	L-Arginine	40	29.2 (28.9–29.4)	1,168 (1095.1–1242.2)	(1)(3)
			Placebo	43	28.8 (28.5–29.1)	1,127 (1047.1–1207.6)	
Riskin 2010	Israel	RCTs	Prebiotic	15	30.3 ± 2.8	1,523 ± 550	(1)(2)(3)(6)
			Placebo	13	28.7 ± 2.9	1,207 ± 447	
Rojas 2012	United States	RCTs	Probiotic	372	32(30–33)	1,530(1253–1750)	(1)(3)(6)
			Placebo	378	32(29–33)	1,516(1129–1750)	
Rouge 2009	France	RCTs	Probiotic	45	28.1 ± 1.9	1,115 ± 251	(1)(2)(3)(6)
			Placebo	49	28.1 ± 1.8	1,057 ± 260	
Samanta 2008	India	RCTs	Probiotic	91	30.12 ± 1.63	1,172 ± 143	(1)(2)(3)
			Placebo	95	30.14 ± 1.59	1,210 ± 143	
Sari 2011	Turkey	RCTs	Probiotic	110	29.5 ± 2.4	1,231 ± 262	(1)(2)(3)
			Placebo	111	29.7 ± 2.4	1,278 ± 282	
Sari 2012	Turkey	RCTs	Probiotic	86	29.7 ± 2.5	1,241 ± 264	(1)(2)
			Placebo	88	29.8 ± 2.3	1,278 ± 273	
Serce 2013	Turkey	RCTs	Probiotic	104	28.7 ± 2.1	1,162 ± 216	(1)(2)(3)(6)
			Placebo	104	28.8 ± 2.2	1,126 ± 232	
Sevastiadou 2011	Greece	RCTs	Glutamine	51	30.85 ± 2.36	1,327 ± 336	(2)
			Placebo	50	30.07 ± 2.47	1,283 ± 346	
Shashidhar 2017	India	RCTs	Probiotic	48	31.2 ± 2.1	1,256 ± 185	(1)(3)(4)(5)(6)
			Placebo	48	31.2 ± 2.1	1,190 ± 208	
Sherman 2016	United States	RCTs	Lactoferrin	59	28 ± 0.85	1,152 ± 206	(1)(2)(3)(5)(6)
			Placebo	60	28 ± 0.85	1,143 ± 220	
Song 2016	China	RCTs	EPO	366	30.39 ± 1.38	1,372 ± 209	(1)(2)(3)
			Placebo	377	30.40 ± 1.46	1,396 ± 239	
Sowden 2022	South Africa	RCTs	Probiotic	100	26–36	750–1,500	(1)(4)(5)
			Placebo	100	26–36	750–1,500	
Stratiki 2007	Greece	RCTs	Probiotic	41	31(27–37)	1,500 (900–1780)	(1)(2)(5)
			Placebo	36	30.5(26–37)	1,500 (700–1900)	
Strus 2018	Poland	RCTs	Probiotic	90	29.73 ± 2.26	1281.24 ± 281.18	(1)(2)(3)
			Placebo	91	29.67 ± 2.32	1350.11 ± 292.18	
Tanjina 2016	United Kingdom	RCTs	Probiotic	52	31.38 ± 0.93	1310.6 ± 110.41	(1)(5)(6)
			Placebo	50	31.68 ± 0.84	1338.0 ± 97.71	
Tarnow-Mordi 2020	Australia	RCTs	Lactoferrin	770	28.4 ± 2.4	1,068 (262)	(1)(2)(3)
			Placebo	771	28.4 ± 2.3	1,063 (261)	
Tewari 2015	India	RCTs	Probiotic	61	<34	<2,500	(1)(2)(3)
			Placebo	59	<34	<2,500	
Thompson 2003	United Kingdom	RCTs	Glutamine	12	27.0 ± 1.7	862 ± 206	(5)
			Placebo	16	27.8 ± 1.7	920 ± 249	
Totsu 2014	Japan	RCTs	Probiotic	153	28.6 ± 2.9	1,016 ± 289	(1)(2)(3)(5)(6)
			Placebo	150	28.5 ± 3.3	998 ± 281	
Turker 2005	Turkey	RCTs	EPO	42	30(24–33)	1,110 (650–1,490)	(1)
			Placebo	51	31(24–33)	1,200 (530–1,495)	
Varaporn 2014	Thailand	RCTs	Probiotic	31	31.0 + 1.82	1250.1 + 179.26	(1)(2)(3)(5)(6)
			Placebo	29	30.59 + 1.76	1207.72 + 199.35	
Vaughn 2003	United States	RCTs	Glutamine	314	27 ± 2	890 ± 200	(2)
			Placebo	335	27 ± 2	900 ± 190	
Wang 2020	China	RCTs	EPO	641	29.7 (28.9–30.9)	1,250 (1100–1,410)	(1)
			Placebo	644	30.0 (29.0–31.0)	1,300 (1100–1,450)	
Wejryd 2018	Sweden	RCTs	Probiotic	68	25.5 ± 1.2	731 ± 129	(1)(2)(3)(5)
			Placebo	66	25.5 ± 1.3	740 ± 148	
Xu 2016	China	RCTs	Probiotic	63	33 + 0.72	1947 ± 54	(2)(5)(6)
			Placebo	62	33 + 1.04	1957 ± 51	
Yeo 2001	Singapore	RCTs	EPO	54	28.2 ± 1.9	988 ± 248	(1)(2)(3)
			Placebo	54	28.3 ± 2.1	988 ± 254	

EPO, erythropoietin; RCT, randomized controlled trial. (1) The incidence of NEC; (2) the incidence of sepsis; (3) the incidence of overall mortality; (4) the time to beginning enteral feeds; (5) the time to full enteral feeds; (6) duration of hospitalization.

### 3.2 Risk of bias and quality assessment

The quality assessment of the included RCTs was conducted using the Cochrane Collaboration’s “Risk of Bias” tool. The risk of bias assessment for the included studies is presented in Table 3.

**Table 3:** Risk of bias of the included randomized controlled trials.

	Sequence generation	Allocation concealment	Blinding	Completeness of data	Selective reporting bias	Other bias
Akin 2014	Simple envelope randomization	Sealed envelope	Double-blind (participant and therapist)	Low risk	Low risk	Low risk
Al-Hosni 2012	Unclear	Unclear	Double-blind (participant/therapist)	Low risk	Low risk	Low risk
Amin 2002	Unclear	Unclear	Double-blind (participant and therapist)	Low risk	Low risk	Low risk
Armanian 2014	Unequal Randomization as 2:1	Unclear	Unclear	Low risk	Low risk	Low risk
Barrington 2016	Computer-generated	Sealed envelope	Double-blind (participant and therapist)	Low risk	Low risk	Low risk
Bierer 2006	Permuted block method	Unclear	Double-blind (participant and therapist)	Low risk	Low risk	Low risk
Bin nun 2005	Unclear	Unclear	Double-blind (participant and therapist)	Low risk	Low risk	Low risk
Braga 2012	Unclear	Sealed envelope	Double-blind (participant and therapist)	Low risk	Low risk	Low risk
Chang 2022	Unclear	Unclear	Unclear	Low risk	Low risk	Low risk
Chaudhuri 2014	Computer-generated	Unclear	Double-blind (participant and therapist)	Low risk	Low risk	Low risk
Chou 2010	Random-number table Sequence	Sealed envelope	Double-blind (participant and therapist)	Low risk	Low risk	Low risk
Costalos 2003	Unclear	Sealed envelope	Double-blind (participant and therapist)	Low risk	Low risk	Low risk
Costeloe 2016	Minimisation algorithm	Sealed envelope	Double-blind (participant and therapist)	Low risk	Low risk	Low risk
Cui 2019	Unclear	Unclear	Double-blind (participant and therapist)	Low risk	Low risk	Low risk
Dallas 1998	Unclear	Unclear	Double-blind (participant and therapist)	Low risk	Low risk	Low risk
Dani 2002	Unclear	Sealed envelope	Double-blind (participant and therapist)	Low risk	Low risk	Low risk
Dekieviet 2014	Unclear	Unclear	Unclear	Low risk	Low risk	Low risk
Dilli 2015	Unclear	Sealed envelope	Double-blind (participant and therapist)	Low risk	Low risk	Low risk
El-Ganzoury 2014	Computer-generated	Sealed envelope	Double-blind (participant and therapist)	Low risk	Low risk	Low risk
El-Shimi 2015	Unclear	Unclear	Unclear	Low risk	Low risk	Low risk
Fauchere 2015	Computer-generated	Sealed envelope	Double-blind (participant and therapist)	Low risk	Low risk	Low risk
Fauchere 2008	Computer-generated	Sealed envelope	Double-blind (participant and therapist)	Low risk	Low risk	Low risk
Fernandez 2012	Random digit table	Sealed envelope	Double-blind (participant and therapist)	Low risk	Low risk	Low risk
Fujii 2006	Unclear	Unclear	Unclear	Low risk	Low risk	Low risk
Griffiths 2018	Computer-generated	Unclear	Double-blind (participant and therapist)	Low risk	Low risk	Low risk
Haiden2004	Unclear	Sealed envelope	Double-blind (participant and therapist)	Low risk	Low risk	Low risk
Hays 2015	Unclear	Unclear	Triple-blind (participant and therapist and assessor)	Low risk	Low risk	Low risk
Hoyos 1999	Unclear	Unclear	Unclear	Low risk	Low risk	Low risk
Jacobs 2013	Unclear	Sealed envelope	Double-blind (participant and therapist)	Low risk	Low risk	Low risk
Juul 2020	Computer-generated	Sealed envelope	Double-blind (participant and therapist)	Low risk	Low risk	Low risk
Kaban 2019	Alternating Randomization technique	Unclear	Double-blind (participant and therapist)	Low risk	Low risk	Low risk
Kanic 2015	Unclear	Unclear	Unclear	Low risk	Low risk	Low risk
Lacey 1996	Unclear	Unclear	Double-blind (participant and therapist)	Low risk	Low risk	Low risk
Lin 2005	Computer-generated	Unclear	Double-blind (participant and therapist)	Low risk	Low risk	Low risk
Lin 2008	Computer-generated	Unclear	Double-blind (participant and therapist)	Low risk	Low risk	Low risk
Lowe 2017	Computer-generated	Unclear	Double-blind (participant and therapist)	Low risk	Low risk	Low risk
Maier 2002	Unclear	Sealed envelope	Double-blind (participant and therapist)	Low risk	Low risk	Low risk
Manzoni 2006	Computer-generated	Unclear	Double-blind (participant and therapist)	Low risk	Low risk	Low risk
Manzoni 2009	Computer-generated	Unclear	Double-blind (participant/therapist)	Low risk	Low risk	Low risk
Manzoni 2014	Computer-generated	Unclear	Double-blind (participant and therapist)	Low risk	Low risk	Low risk
Mihatsch 2010	Computer-generated	Sealed envelope	Double-blind (participant and therapist)	Low risk	Low risk	Low risk
Modi 2010	Unclear	Unclear	Unclear	Low risk	Low risk	Low risk
Mohamad 2011	Computer-generated	Unclear	Double-blind (participant and therapist)	Low risk	Low risk	Low risk
Hosseini 2019	Unclear	Unclear	Double-blind (participant and therapist)	Low risk	Low risk	Low risk
Nandhini 2015	Computer-generated	Sealed envelope	Double-blind (participant and therapist)	Low risk	Low risk	Low risk
Natalucci 2016	Unclear	Unclear	Double-blind (participant and therapist)	Low risk	Low risk	Low risk
Obladen 1991	Unclear	Sealed envelope	Double-blind (participant and therapist)	Low risk	Low risk	Low risk
Ochoa 2020	Unclear	Sealed envelope	Double-blind (participant and therapist)	Low risk	Low risk	Low risk
O’Gorman 2015	Computer-generated	Sealed envelope	Triple-blind (participant and therapist and assessor)	Low risk	Low risk	Low risk
Ohls 2013	Computer-generated	Sealed envelope	Double-blind (participant and therapist)	Low risk	Low risk	Low risk
Ohls 2001	Computer-generated	Sealed envelope	Double-blind (participant and therapist)	Low risk	Low risk	Low risk
Ohls 2004	Unclear	Unclear	Unclear	Low risk	Low risk	Low risk
Omar 2020	Unclear	Unclear	Double-blind (participant and therapist)	Low risk	Low risk	Low risk
Oncel 2013	Computer-generated	Sealed envelope	Double-blind (participant and therapist)	Low risk	Low risk	Low risk
Shannon 1995	Unclear	Unclear	Double-blind (participant and therapist)	Low risk	Low risk	Low risk
Demirel 2013	Computer-generated	Sealed envelope	Double-blind (participant and therapist)	Low risk	Low risk	Low risk
Dutta 2015	Unclear	Sealed envelope	Double-blind (participant and therapist)	Low risk	Low risk	Low risk
Güney-Varal 2017	Unclear	Unclear	Unclear	Low risk	Low risk	Low risk
Singh S 2017	Unclear	Unclear	Double-blind (participant and therapist)	Low risk	Low risk	Low risk
Patole 2014	Unclear	Unclear	Double-blind (participant and therapist)	Low risk	Low risk	Low risk
Peltoniemi 2017	Random number table	Sealed envelope	Double-blind (participant and therapist)	Low risk	Low risk	Low risk
Poindexter 2004	Unclear	Unclear	Double-blind (participant and therapist)	Low risk	Low risk	Low risk
Polycarpou 2013	Unclear	Sealed envelope	Double-blind (participant and therapist)	Low risk	Low risk	Low risk
Riskin 2010	Unclear	Sealed envelope	Double-blind (participant and therapist)	Low risk	Low risk	Low risk
Rojas 2012	Computer-generated	Sealed envelope	Double-blind (participant and therapist)	Low risk	Low risk	Low risk
Rouge 2009	Unclear	Unclear	Double-blind (participant and therapist)	Low risk	Low risk	Low risk
Samanta 2008	Random number table	Unclear	Double-blind (participant and therapist)	Low risk	Low risk	Low risk
Sari 2011	Computer-generated	Sealed envelope	Double-blind (participant and therapist)	Low risk	Low risk	Low risk
Sari 2012	Computer-generated	Sealed envelope	Double-blind (participant and therapist)	Low risk	Low risk	Low risk
Serce 2013	Computer-generated	Sealed envelope	Double-blind (participant and therapist)	Low risk	Low risk	Low risk
Sevastiadou 2011	Unclear	Unclear	Double-blind (participant and therapist)	Low risk	Low risk	Low risk
Shashidhar 2017	Computer-generated	Sealed envelope	Double-blind (participant and therapist)	Low risk	Low risk	Low risk
Sherman 2016	Unclear	Unclear	Double-blind (participant and therapist)	Low risk	Low risk	Low risk
Song 2016	Computer-generated	Sealed envelope	Double-blind (participant and therapist)	Low risk	Low risk	Low risk
Sowden 2022	Unclear	Unclear	Double-blind (participant and therapist)	Low risk	Low risk	Low risk
Stratiki 2007	Unclear	Unclear	Double-blind (participant and therapist)	Low risk	Low risk	Low risk
Strus 2018	Unclear	Unclear	Double-blind (participant and therapist)	Low risk	Low risk	Low risk
Tanjina 2016	Unclear	Unclear	Double-blind (participant and therapist)	Low risk	Low risk	Low risk
Tarnow-Mordi 2020	Computer-generated	Sealed envelope	Double-blind (participant and therapist)	Low risk	Low risk	Low risk
Tewari 2015	Unclear	Unclear	Double-blind (participant and therapist)	Low risk	Low risk	Low risk
Thompson 2003	Unclear	Unclear	Double-blind (participant and therapist)	Low risk	Low risk	Low risk
Totsu 2014	Computer-generated	Unclear	Double-blind (participant and therapist)	Low risk	Low risk	Low risk
Turker 2005	Unclear	Unclear	Unclear	Low risk	Low risk	Low risk
Varaporn 2014	Unclear	Unclear	Double-blind (participant and therapist)	Low risk	Low risk	Low risk
Vaughn 2003	Unclear	Sealed envelope	Double-blind (participant and therapist)	Low risk	Low risk	Low risk
Wang 2020	Unclear	Unclear	Double-blind (participant and therapist)	Low risk	Low risk	Low risk
Wejryd 2018	Computer-generated	Sealed envelope	Double-blind (participant and therapist)	Low risk	Low risk	Low risk
Xu 2016	Unclear	Unclear	Double-blind (participant and therapist)	Low risk	Low risk	Low risk
Yeo 2001	Unclear	Unclear	No-blind	Low risk	Low risk	Low risk

### 3.3 Evidence network

**Figure 2:**
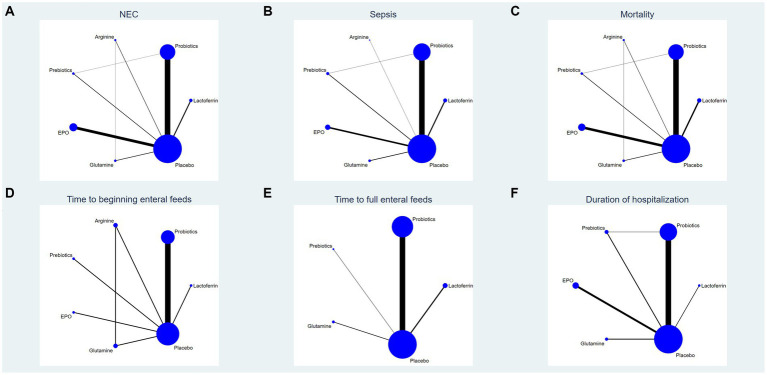
Network analysis of eligible comparison for **(A)** the incidence of NEC, **(B)** the incidence of sepsis, **(C)** the incidence of overall mortality, **(D)** time to beginning enteral feeds, **(E)** time to full enteral feeds and **(F)** duration of hospitalization. The size of each node represents the number of participants, while the thickness of the line represents the number of studies directly comparing the two interventions.

This study encompassed 6 drugs (7 interventions), including lactoferrin, probiotics, prebiotics, glutamine, arginine, erythropoietin and placebo. Figure 2 represents the evidence network, where the lines denote direct comparisons between two directly related interventions. Interventions lacking direct connections are compared indirectly through the NMA. The width of the lines reflects the number of trials, while the size of the nodes corresponds to the total sample size across multiple treatments.

### 3.4 Inconsistency test

**Figure 3:**
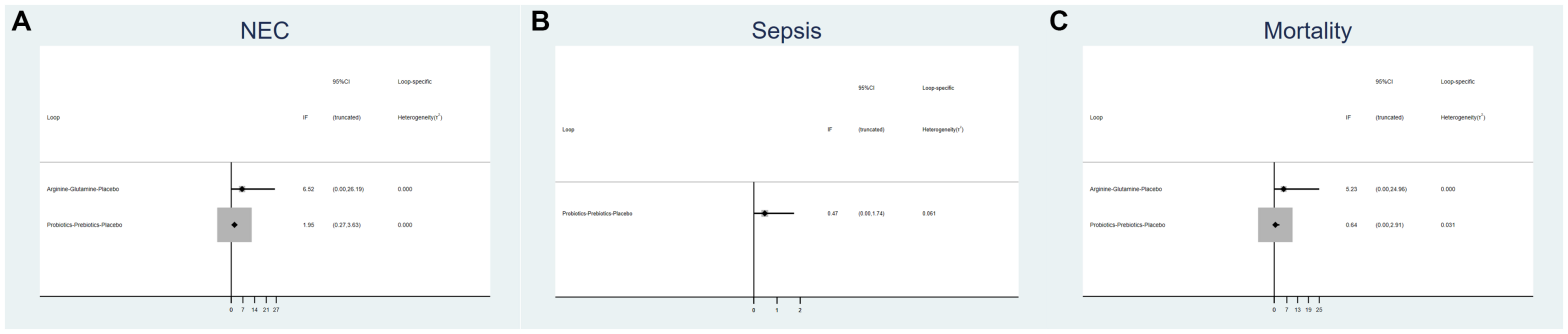
Inconsistency plot of eligible comparison for **(A)** the incidence of NEC, **(B)** the incidence of sepsis and **(C)** the incidence of overall mortality.

Figure 3 displays an inconsistency plot designed to assess heterogeneity among studies within the closed loops of the NMA. There were 5 closed loops for the primary outcomes including the incidence of NEC, NEC-associated sepsis and overall mortality, with IF ranging from 0.47 to 6.52. Most of these closed loops had 95% CIs that contained 0, and only one closed loops of probiotics-prebiotics-placebo had 95% CIs approaching 0. Overall, these results suggest that the data exhibited consistency.

### 3.5 NMA results

#### 3.5.1 Primary outcomes

##### 3.5.1.1 The incidence of NEC

**Figure 4:**
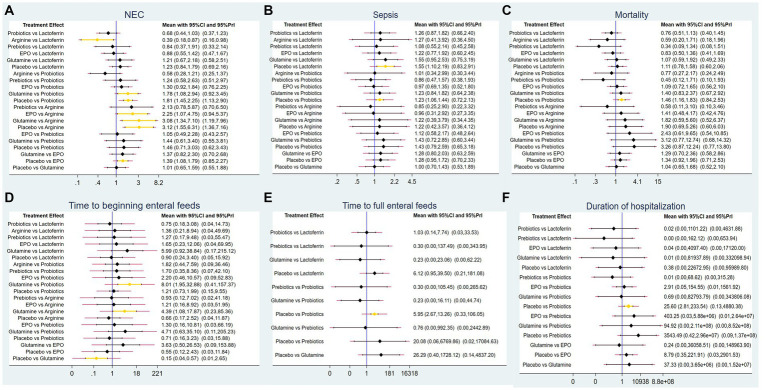
Forest plots for **(A)** the incidence of NEC, **(B)** the incidence of sepsis, **(C)** the incidence of overall mortality, **(D)** time to beginning enteral feeds, **(E)** time to full enteral feeds and **(F)** duration of hospitalization.

A total of 83 RCTs with 25,359 neonates reported the incidence of NEC after treatment, involving interventions of probiotics, prebiotics, glutamine, lactoferrin, EPO, arginine, and placebo. The results of the NMA revealed the following findings regarding the incidence of NEC: arginine therapy was associated with lower incidence of NEC compared lactoferrin (RR = 0.39, 95%CI: 0.18, 0.87), EPO (RR = 2.25, 95%CI: 1.07, 4.75), glutamine (RR = 3.08, 95%CI: 1.34, 7.10) and placebo (RR = 3.12, 95%CI: 1.55, 6.31). Probiotics therapy was associated with a lower incidence of NEC compared glutamine (RR = 1.78, 95%CI: 1.08, 2.94) and placebo (RR = 1.81, 95%CI: 1.45, 2.25). Other comparisons did not yield statistically significant differences (Figure 4A).

**Figure 5:**
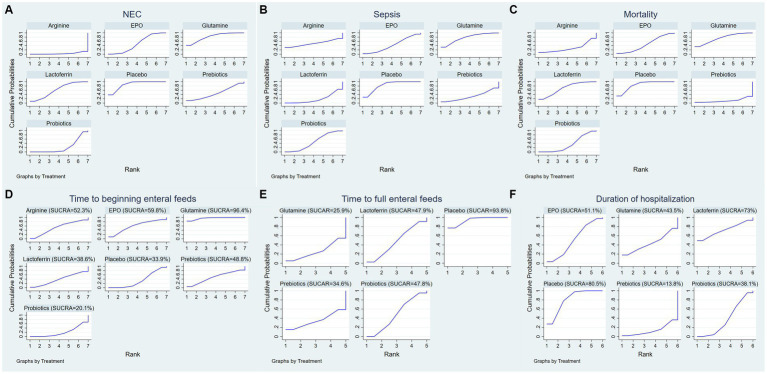
Surface under the cumulative ranking (SUCRA) for **(A)** the incidence of NEC, **(B)** the incidence of sepsis, **(C)** the incidence of overall mortality, **(D)** time to beginning enteral feeds, **(E)** time to full enteral feeds and **(F)** duration of hospitalization.

A ranking graph illustrating the distribution of probabilities for NEC is presented in Figure 5A. The SUCRA rankings for the incidence of NEC were as follows: arginine (3.2%) < probiotics (22.2%) < prebiotics (45.8%) < EPO (48.5%) < lactoferrin (61.7%) < glutamine (81.6%) < placebo (87.1%), which suggests that arginine is associated with the lowest probability of developing NEC while placebo has the lowest effect. Therefore, the efficacy in reducing the incidence of NEC was ranked from best to worst as follows: arginine, probiotics, prebiotics, EPO, lactoferrin, glutamine, and placebo.

##### 3.5.1.2 The incidence of NEC-associated sepsis

A total of 62 RCTs involving 20,994 neonates reported the incidence of post-treatment sepsis. The results of the NMA revealed that lactoferrin (RR = 1.55, 95% CI: 1.10, 2.19) and probiotics (RR = 1.23, 95% CI: 1.06, 1.44) had a higher effect on NEC-associated sepsis compared to placebo. Other comparisons did not yield statistically significant differences (Figure 4B).

A ranking graph illustrating the distribution of probabilities for NEC-associated sepsis is presented in Figure 5B. The SUCRA rankings for the incidence of NEC-associated sepsis were as follows: lactoferrin (18.7%) < prebiotics (31.3%) < EPO (42.9%) < probiotics (46.7%) < arginine (50.5%) < glutamine (77%) < placebo (83%), suggesting that lactoferrin was associated with the lowest probability of developing NEC-associated sepsis while placebo had the lowest effect. Therefore, the efficacy in reducing the incidence of NEC-associated sepsis was ranked from best to worst as follows: lactoferrin, prebiotics, EPO, probiotics, arginine, glutamine, and placebo.

##### 3.5.1.3 The incidence of overall mortality

Sixty-two RCTs involving 20,438 neonates reported the incidence of overall mortality. The results of the NMA revealed that probiotics exhibited a lower incidence of overall mortality compared to placebo (RR = 1.46, 95%CI: 1.16, 1.83). Other comparisons did not yield statistically significant differences (Figure 4C).

A ranking graph illustrating the distribution of probabilities for overall mortality is presented in Figure 5C. The SUCRA rankings for the incidence of overall mortality were as follows: prebiotics (11.1%) < arginine (28.5%) < probiotics (35.3%) < EPO (45.9%) < lactoferrin (69.4%) < glutamine (74.9%) < placebo (84.8%), suggesting that prebiotics was associated with the lowest overall mortality while placebo had the lowest effect. Therefore, the efficacy in reducing the incidence of overall mortality was ranked from best to worst as follows: prebiotics, arginine, probiotics, EPO, lactoferrin, glutamine, and placebo.

#### 3.5.2 Secondary outcomes

##### 3.5.2.1 Time to beginning enteral feeds

Only 11 RCTs involving 2,144 neonates reported the time to beginning enteral feeds. The results of the NMA revealed the following findings: glutamine demonstrated a longer time compared to probiotics (WMD = 8.01, 95%CI: 1.95, 32.88), arginine (WMD = 4.39, 95%CI: 1.08, 17.87) and placebo (WMD = 0.15, 95%CI: 0.04, 0.57). Other comparisons did not yield statistically significant differences (Figure 4D).

A ranking graph illustrating the distribution of probabilities for the time to beginning enteral feeds is presented in Figure 5D. Based on the SUCRA, probiotics had the lowest SUCRA rank, indicating the lowest probability of the time to beginning enteral feeds, while glutamine had the highest probability. The SUCRA rankings for time to beginning enteral feeds were as follows: probiotics (20.1%) < placebo (33.9%) < lactoferrin (38.6%) < prebiotics (48.8%) < arginine (52.3%) < EPO (59.8%) < glutamine (96.4%). Therefore, the efficacy in shortening the time to beginning enteral feeds was ranked from best to worst as follows: probiotics, placebo, lactoferrin, prebiotics, arginine, EPO, and glutamine.

##### 3.5.2.2 Time to full enteral feeds

A total of 27 RCTs with 5,916 neonates reported the time to full enteral feeds, involving five interventions including glutamine, prebiotics, probiotics, lactoferrin, and placebo. The NMA results revealed the following findings: probiotics demonstrated a shorter time to full enteral feeds compared to placebo (WMD = 5.95, 95%CI: 2.67, 13.26). Other comparisons did not yield statistically significant differences (Figure 4E).

A ranking graph illustrating the distribution of probabilities for the time to full enteral feeds is presented in Figure 5E. Based on the SUCRA, glutamine had the lowest SUCRA rank, indicating the lowest probability of the time to full enteral feeds, while placebo had the highest probability. The SUCRA rankings for time to full enteral feeds were as follows: glutamine (25.9%) < prebiotics (34.6%) < probiotics (47.8%) < lactoferrin (47.9%) < placebo (93.8%). Therefore, the efficacy in shortening the time to full enteral feeds was ranked from best to worst as follows: glutamine, prebiotics, probiotics, lactoferrin, and placebo.

##### 3.5.2.3 Duration of hospitalization

A total of 34 RCTs with 9,642 neonates reported duration of hospitalization, involving six interventions, including lactoferrin, probiotics, prebiotics, glutamine, EPO, and placebo. The NMA results revealed the following: probiotics demonstrated a shorter duration of hospitalization compared to placebo (WMD = 25.6, 95%CI: 2.81, 233.54). Other comparisons did not yield statistically significant differences (Figure 4F).

A ranking graph illustrating the distribution of probabilities for duration of hospitalization is presented in Figure 5F. Based on the SUCRA, prebiotics had the lowest SUCRA rank, indicating the lowest probability of duration of hospitalization, while placebo had the highest probability. The SUCRA rankings for duration of hospitalization were as follows: prebiotics (13.8%) < probiotics (38.1%) < glutamine (43.5%) < EPO (51.1%) < lactoferrin (73%) < placebo (80.5%). Therefore, the efficacy in shortening duration of hospitalization was ranked from best to worst as follows: prebiotics, probiotics, glutamine, EPO, lactoferrin, and placebo.

### 3.6 Publication bias

**Figure 6:**
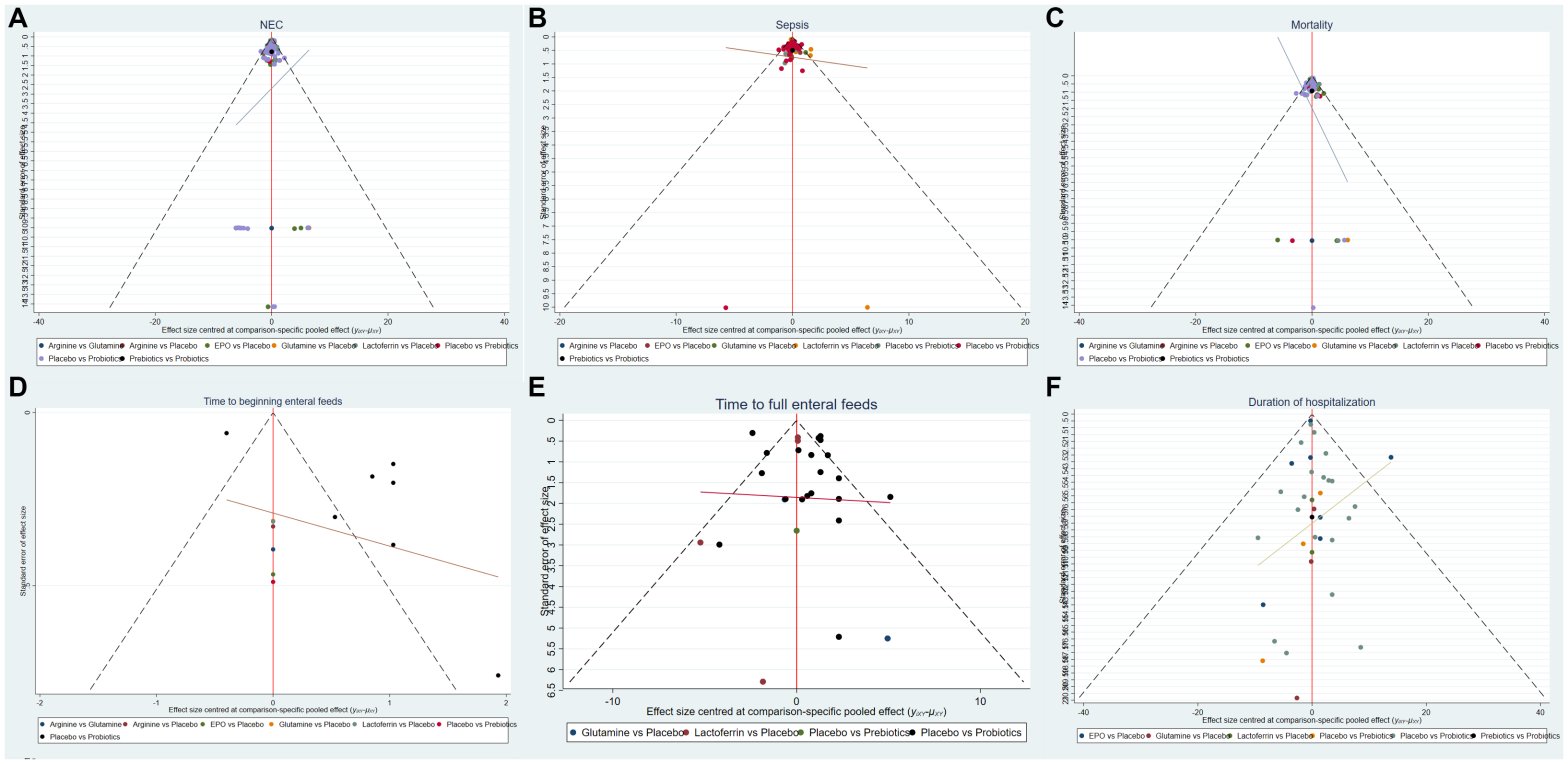
Funnel plots of **(A)** the incidence of NEC, **(B)** the incidence of sepsis, **(C)** the incidence of overall mortality, **(D)** time to beginning enteral feeds, **(E)** time to full enteral feeds and **(F)** duration of hospitalization.

Based on the outcomes observed for the incidence of NEC, NEC-associated sepsis, overall mortality, time to beginning enteral feeds, time to full enteral feeds and duration of hospitalization, NMA showed that the corrected funnel plots were generated to assess publication bias and potential small sample effects. The analysis revealed that most data points were well-distributed within the funnel plot, displaying relative symmetry on both sides. Additionally, the regression line closely paralleled the X-axis, indicating minimal likelihood of publication bias or small sample effects (Figure 6).

## 4 Discussion

NEC continues to be one of the most severe acute GI afflictions in preterm and low-birth-weight infants (30). However, its precise etiology and pathogenesis are still not fully understood (31). Key factors implicated in NEC include intestinal mucosal barrier dysfunction, ischemia–reperfusion injury, inflammatory responses, and an imbalance in gut microbiota (32). Without effective treatments for NEC, research has shifted toward prevention strategies. Early initiation of breastfeeding has shown to be beneficial, particularly in preterm and low birth weight infants (5, 6, 33). However, the susceptibility to NEC is paradoxically increased (33–35) due to dysfunctional suckling and swallowing, GI reflux, and impaired motor coordination (36–38). As a result, parenteral nutrition is commonly initiated in these infants. The search for alternative NEC prevention methods has led to the discovery that probiotics, prebiotics, arginine, lactoferrin, EPO, and glutamine have significant roles in the primary prevention of NEC (17, 19, 20). With advancing insights into the pathogenesis of NEC, new avenues for prevention and treatment are continually being explored.

This study integrates data from 89 RCTs on six interventions (including probiotics, prebiotics, arginine, lactoferrin, EPO, and glutamine), utilizing NMA to evaluate their impact on NEC incidence, NEC-associated sepsis and mortality, and to rank their probabilities of efficacy. NMA indicated the following ranking from most to least effective in decreasing the incidence of NEC in preterm infants: arginine, probiotics, prebiotics, erythropoietin, lactoferrin, glutamine, placebo; for the reduction of NEC-associated sepsis events: lactoferrin, prebiotics, erythropoietin, probiotics, arginine, glutamine, placebo; and for the reduction of overall mortality: prebiotics, arginine, probiotics, erythropoietin, lactoferrin, glutamine, placebo. The ranking for time to beginning enteral feeds was: probiotics, placebo, lactoferrin, prebiotics, arginine, erythropoietin, glutamine; for time to full enteral feeds: glutamine, prebiotics, probiotics, lactoferrin, placebo; and for hospital stay duration: prebiotics, probiotics, glutamine, erythropoietin, lactoferrin, placebo. A comprehensive analysis of these six outcomes suggests an overall clinical efficacy ranking from most to least effective for the aforementioned drugs as follows: arginine, probiotics, prebiotics, lactoferrin, erythropoietin, glutamine, and placebo.

Intestinal microcirculatory perfusion is predominantly regulated by nitric oxide (NO), a vasodilator synthesized via the activity of endothelial nitric oxide synthase (eNOS) (7). Upon entry of harmful bacteria into the circulation, expression levels of eNOS are suppressed. Decreased plasma NO levels can lead to significant vasoconstriction, disrupts intestinal perfusion and result in hypoxia, a hallmark of necrosis seen in NEC. To boost eNOS activity, Moreira et al. (39) incorporated arginine into their research, an amino acid precursor to NO that is crucial for preventing tissue injury (40). A deficiency in endogenous arginine synthesis can restrict NO production and impair vasodilation in the postprandial intestinal circulation. Chen et al. (41) discovered that arginine supplementation increases blood flow within the intestinal microvasculature and can prevent NEC, whereas arginine antagonists may intensify the condition. The findings of the present study further indicate that arginine significantly reduces the incidence of NEC in premature infants, which aligns with the recent findings by Wang et al. (42). Moreover, arginine demonstrates a substantial advantage in decreasing the incidence of sepsis and overall mortality.

Compared to placebo, lactoferrin showed a statistically significant difference in efficacy in reducing the incidence of NEC and NEC-associated sepsis. Acccording to probability ranking, lactoferrin is the most effective intervention in decreasing the incidence of NEC-associated sepsis, outperforming other measures. These findings largely align with prior meta-analytic conclusions (43, 44). The broad-spectrum antimicrobial effects of lactoferrin are likely due to its multiple mechanisms of action, including cell membrane disruption, iron sequestration, immune modulation, and direct antimicrobial activity, which collectively inhibit the growth of bacteria, fungi, and viruses (45). This contributes to reducing the incidence of advanced NEC stages, specifically stages II and III NEC (44). However, there is a discrepancy with the findings of Gao’s study (46), potentially due to limited study inclusion and a small sample size.

Prebiotics showed superior efficacy in reducing overall mortality and hospital stay of NEC patients. Prebiotics naturally present in breast milk, comprising over 200 varieties of human milk oligosaccharides (HMOs) (47). These prebiotics promote the proliferation of beneficial microbes such as Bifidobacteria and Lactobacilli. Their life-saving potential is likely due to the prevention of pathogen colonization and the unchecked growth of opportunistic pathogens (48). Furthermore, prebiotics enhance gut motility and permeability in preterm infants, thus improving intestinal epithelium integrity. The synergistic effects of pathogen inhibition and the prevention of their adherence to the epithelial surface may bolster the resistance of preterm infants to endogenous infections (49, 50). This study also corroborates that probiotics expedite the initiation of postnatal enteral feeding. Aligning with the findings by Athalye-Jape et al. (51), this may be attributed to the promotion of GI maturity and motility through the extension of intestinal transit time, acceleration of gastric emptying, and augmentation of mesenteric arterial blood flow post-probiotic administration.

The present study has some limitations. First, only English-language literature was included. Secondly, the interpretability of findings is restricted due to inadequate details on randomization methods and allocation concealment in many trials. Thirdly, an economic analysis was not performed.

Despite these limitations, the key strengths of this paper are: (1) an expanded evaluation of interventional drugs based on prior research, offering a broader comparison of clinical efficacies for preventing NEC in preterm infants, with results reflecting the most comprehensive current evidence; (2) inclusion of 89 RCTs, addressing the previous meta-analyses limitations of the limited study scope and sample size, thus providing a more robust evidence base.

## 5 Conclusion

Existing literature highlights arginine as the most efficacious pharmacological agent in preventing NEC in preterm infants. It has been shown to effectively lower the rates of NEC, septicemia, and mortality, warranting its recommendation as the first-line clinical intervention. Following this, probiotics are recommended as a second option.

## Data availability statement

The original contributions presented in the study are included in the article/supplementary material, further inquiries can be directed to the corresponding author.

## Author contributions

JC: Conceptualization, Data curation, Formal analysis, Funding acquisition, Investigation, Methodology, Project administration, Resources, Software, Supervision, Validation, Visualization, Writing – original draft, Writing – review & editing. XC: Data curation, Methodology, Resources, Supervision, Writing – original draft, Writing – review & editing. XH: Investigation, Resources, Supervision, Visualization, Writing – review & editing. JL: Resources, Supervision, Writing – review & editing. QY: Resources, Supervision, Writing – review & editing.
